# The Principles of House-Drainage

**Published:** 1886-04

**Authors:** 


### BOOK REVIEWS.

THE PRINCIPLES OF HOUSE-DRAINAGE. *By* J. PICKERING PUTNAM. *Boston:* TICKNOR & CO.; *Chicago:* JANSEN, MCCLURG & CO. *pp. 125, with illustrations.*

This is a practical little book, written by an architect who has evidently made a full study of the subject. It is intended for the general reader as much as for those engaged in the specialties of building, and contains excellent descriptions and explanations.

It must be said, however, that the author is interested in the sale of various plumbing fixtures, invented by himself, and he has probably given too much space to a discussion of these.

